# Concreteness/abstractness ratings for two-character Chinese words in MELD-SCH

**DOI:** 10.1371/journal.pone.0232133

**Published:** 2020-06-22

**Authors:** Xu Xu, Jiayin Li

**Affiliations:** Shanghai Jiao Tong University, Shanghai, China; Centre National de la Recherche Scientifique, FRANCE

## Abstract

The concreteness-abstractness continuum is considered a primary dimension in the representation of semantic networks. Its theoretical importance and clinical significance are widely acknowledged. To assist and enhance future research, this study collected and evaluated concreteness/abstractness ratings for 9,877 two-character Chinese words retrieved from the MEga study of Lexical Decision in Simplified CHinese (MELD-SCH, Tsang et al, 2018). The ratings were validated through comparisons with previous rating studies on concreteness and imageability of smaller word samples. Relations of word concreteness with word frequency, age-of-acquisition, and efficiency of lexical processing were also examined. These ratings provide an additional dimension of information to two-character words in the database MELD-SCH, permitting not only more comprehensive research on the Chinese language, but also cross-language investigation of the concreteness effect between Chinese and other languages such as English and Dutch where a large database of concreteness ratings is also available.

## 1. Introduction

**Fig 1:**
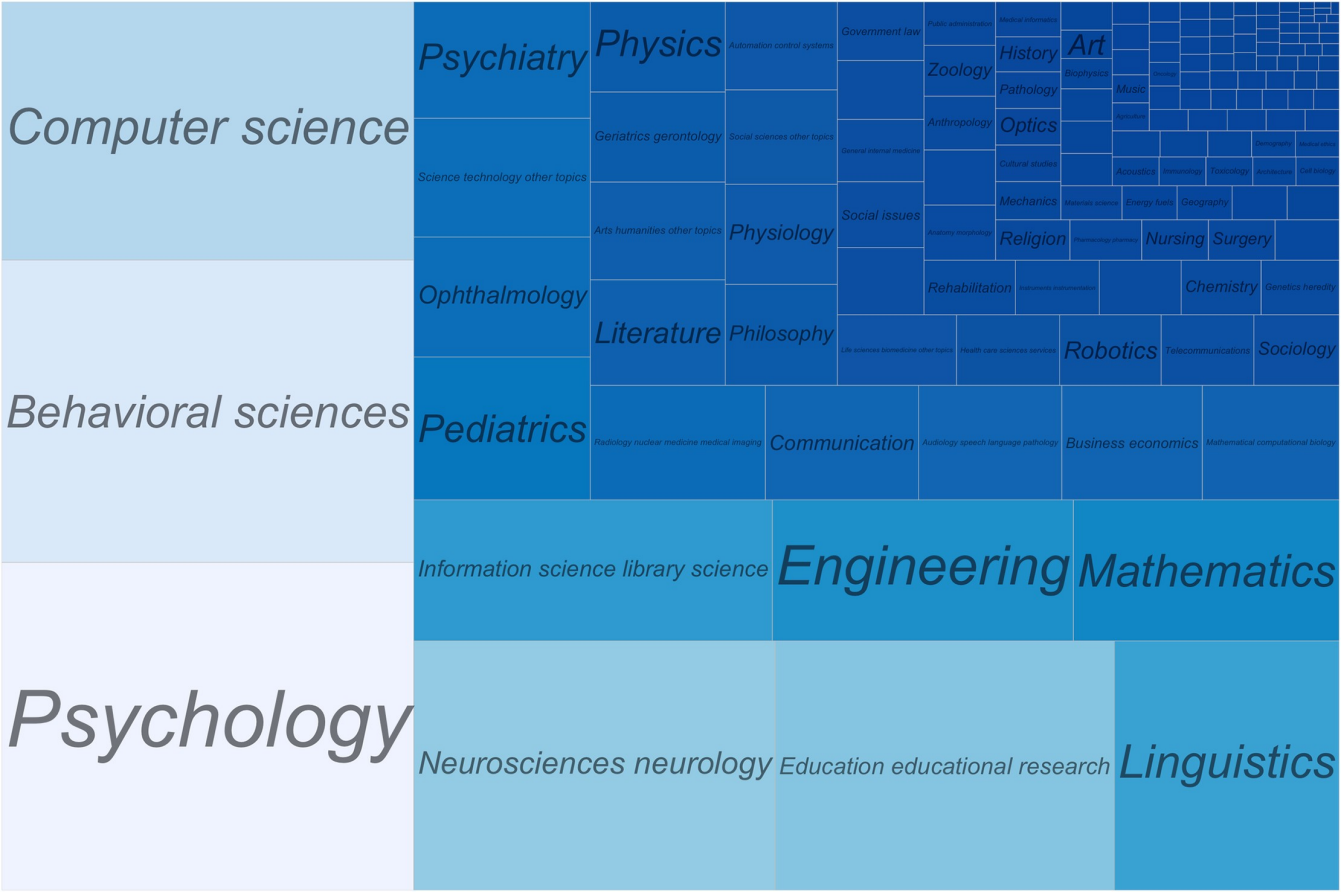
Publications on abstract and concrete concepts or words cover a wide range of disciplines. sizes of the blocks roughly correspond to proportions of publications in different disciplines.

**Fig 2:**
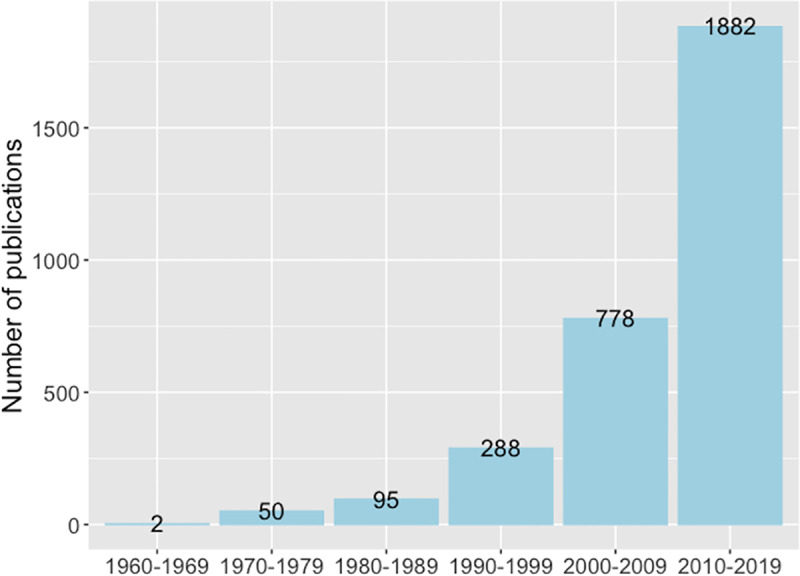
Numbers of publications on abstract and concrete concepts or words plotted over six decades.

Concreteness, or abstractness, has long been investigated in research literatures such as concept representation, language processing, brain functional networks, and others. A “topic” search on the Web of Science returned over 3,000 research articles on abstract and concrete concepts or words. These studies covered a wide range of disciplines including psychology, computer science, neuroscience, linguistics, and education (Fig 1). Moreover, attention directed to this topic appears to become intensified over the years, reflecting an ever-increasing level of interest among researchers. Fig 2 plots the numbers of articles of the search results from the Web of Science over six decades starting from the 1960s to the year of 2019.

Parallel to the amount of research published on this topic, a significant number of theories, models, and proposals have been brought forward in order to explain the distinctions between concrete and abstract conceptual domains or the effects of concreteness versus abstractness. Among them, the dual-code theory [1] and the contextual availability theory [2,3] are probably the most frequently cited. The dual-code theory posits that the meanings of concrete words are represented in both a verbal system and an imagery system, which renders them easier to process relative to abstract words represented solely in the verbal system [1]. In contrast, the contextual availability theory states that the processing advantage of concrete words over abstract words stems from the support of readily available contextual information for concrete words [2]. Diverging from these two theories, the conceptual metaphor theory hypothesizes that representations within the abstract domain are built through conceptual mapping on the basis of representations within the concrete domain [4,5]. Furthermore, there are also theories that attempt to pinpoint the unique features characteristic of abstract concepts, such as situational and introspective experiences [6, 7] and affective experiences [8].

Clinical implications of the concreteness and abstractness effects have also drawn significant attention. Selective impairments of concrete versus abstract words have been documented since the 1970s [9]. For example, there are reports that both patients of Parkinson’s disease without mild cognitive impairment [10] and patients of schizophrenia spectrum disorders [11] display selective impairments of concrete action verbs (e.g., swim, push) in language production tasks. Moreover, research on Alzheimer’s disease (AD) and semantic dementia (SD) suggests that processing concrete and abstract words implicate partially dissociable brain networks. Whereas patients of AD show earlier deterioration of abstract words (e.g., reason, friendship) relative to concrete words (e.g., chair, box), patients of SD appear to exhibit a reversed pattern [12, 13, 14]. Studies with post-stroke patients corroborate the dissociation of neural representations between abstract and concrete words. Lesion in ventrolateral prefrontal cortex has been linked to impairment of abstract words [15]. So have lesions in left temporal superior gyrus, left middle gyrus, and insula [16]. Specific to verbs, damages to left posterior temporal and occipital regions [17] and frontal white matter [18] seem to be associated with greater impairment of concrete action verbs (e.g., throw) relative to abstract ones (e.g., excuse).

Given its theoretical and clinical significance, studies assessing word concreteness can be found for many different languages. Specifically, concreteness ratings have been published for 40,000 English words [19] and 30,000 Dutch words [20]. In addition to these large-scale databases, collections of concreteness ratings for over 1,000 words are also available for German (2,654 nouns; [21]), French (1,659 nouns, verbs, adjectives, adverbs, and pronouns; [22]), Italian (1,121 nouns, adjectives, and verbs; [23]), Spanish (1,400 nouns, verbs, adjectives, adverbs, and interjections; [24]), Portuguese (3,800 content words; [25]), Polish (4,900 nouns, verbs, adjectives, adverbs, and other; [26]), Croatian (3,022 words; [27]), and Indonesian (1,490 words; [28]).

In Mandarin Chinese, a word may consist of one character or multiple characters. For single-character words, [29] published concreteness ratings for 2,423 words, including nouns, verbs, and adjectives. Wang, Huang, Zhou, and Cai [30] presented ratings for 400 single-character words. For two-character words, Yao, Wu, Zhang, and Wang [31] reported ratings for 1,100 words, including nouns, verbs, and adjectives. Yee [32] collected ratings for a smaller set of two-character words (292 nouns).

Two-character words account for approximately 70% of the Chinese lexicon [33, 34]. The existing collections of concreteness ratings for two-character Chinese words are therefore limited in scale. The present study aimed to obtain assessments for a larger set of two-character words. In addition, the two aforementioned studies that provided concreteness ratings for two-character words had a limited set of words in common (n = 26), and the two sets of ratings for these common words were not in line with one another, *r* (26) = .04, *p* = .83, which makes it unfeasible for them to validate or supplement each other [31, 32].

**Fig 3:**
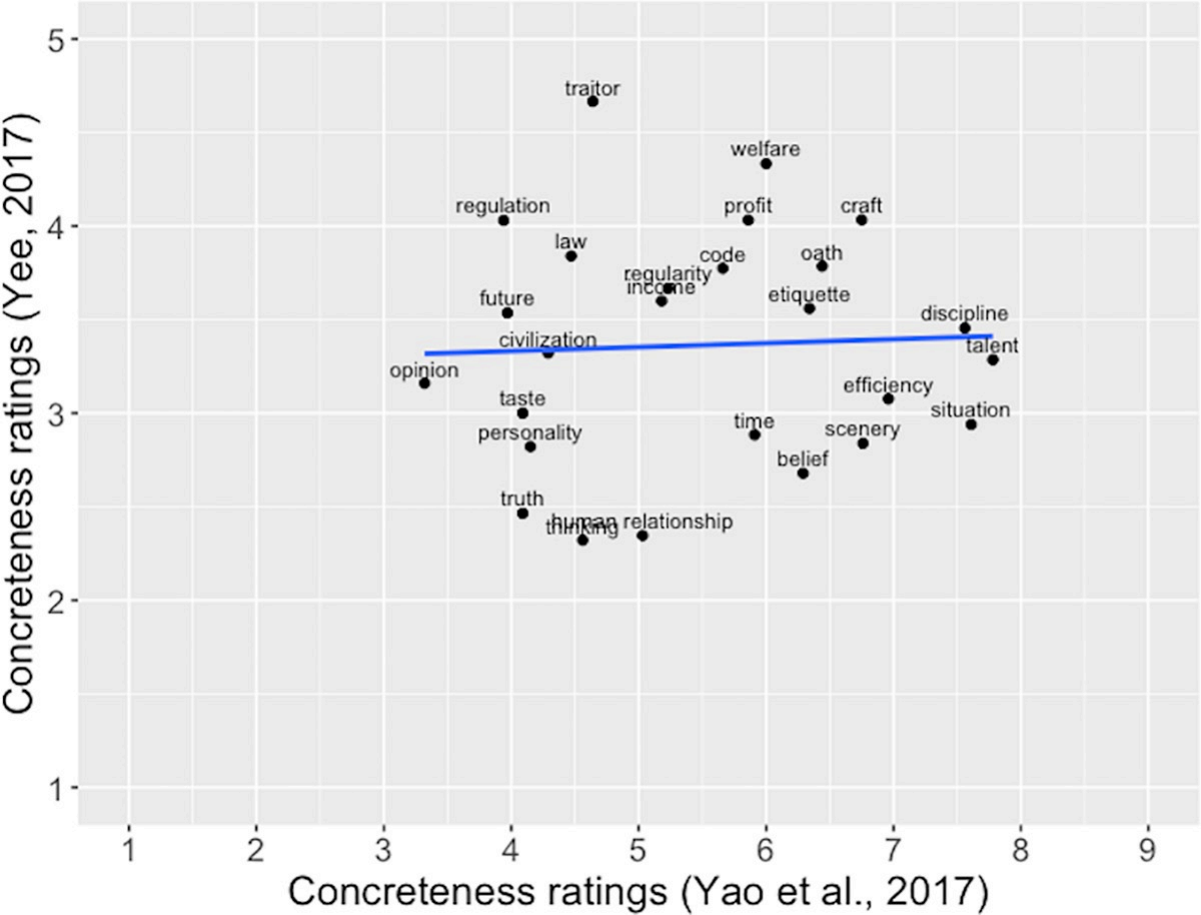
**Correlation of concreteness ratings by Yao et al. [31] and Yee [32], *r* = .04, *p* = .83**. (in Yao et al. [31], “1” represented “very abstract”, and “9” represented “very concrete”; in Yee [32], “1” represented “very abstract”, and “5” represented “very concrete”.).

Fig 3 illustrates ratings of these common words in the two studies. Whereas Yao et al. [31] utilized a 9.0 scale (1 = “very abstract” and 9 = “very concrete”), Yee [32] employed a 5.0 scale (1 = “very abstract” and 5 = “very concrete”). The 26 common words had mean concreteness ratings clustering largely near the center of both scales. That is, these words might feel ambiguous to raters of both studies, neither definitively abstract nor definitively concrete. (The original 26 Chinese words with their English translations are presented as Appendix.) As a result, variability in raters’ perceived concreteness/abstractness about these words must have been substantial, which possibly explains the lack of agreement between the two sets of ratings about these words. The present study with a larger set of two-character words therefore also expected to help validate the existing concreteness ratings of two-character Chinese words.

## 2. Method

### 2.1 Participants

APA ethical standards were followed in the conduct of this study. Participants voluntarily and anonymously responded to and filled out the questionnaires we posted online. They were free to withdraw at any point during this process. At the end of the study, they received monetary compensation for participation. A total of 1,140 participants completed the online questionnaires. All were native speakers of Mandarin Chinese, and spent most of the first seven years of their lives in mainland China.

**Fig 4:**
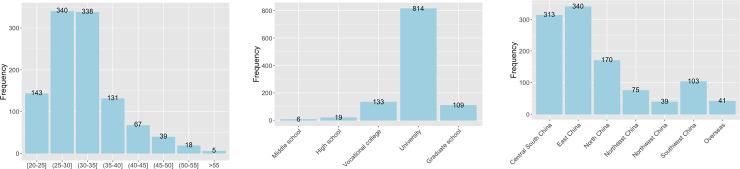
a. Age distribution of the participants. b. Education level distribution of the participants. c. Geographical distribution of the participants.

Based on data screening criteria (see Results section), 59 participants were excluded from further analysis. There were therefore 1,081 participants (51.3% women) included to calculate mean concreteness ratings. Their age ranged from 20 to 65 (Mean = 32.31, SD = 6.83). Education level ranged from middle school to graduate school, with 97.7% college-level or above college-level education. Fig 4A and 4B present frequency distributions of age and education level, respectively. The majority of the participants were from a wide range of geographical regions in China, and a small portion of them resided overseas at the time of the study. Fig 4C shows geographical distribution of the participants.

### 2.2 Word sample

The word sample came from the MEga study of Lexical Decision conducted with Simplified CHinese characters and words (MELD-SCH; [35]). For the purpose of their study, Tsang et al. [35] retrieved 20,000 one- to four-character words from the SUBTLEX-CH corpus [36]. After removing proper nouns, their word sample contained 12,578 words, including 10,022 two-character words. As two-character words account for approximately 70% Chinese lexicon [33, 34] and research on the Chinese language typically samples two-character words as stimuli, we focused the present rating study on this category of words. From Tsang et al.’s sample of two-character words, we further removed the following: 1) words with r-ending retroflexion, e.g., 活儿 ‘duty’, where the second character儿 functions as a phonological suffix to a word and is usually not written out [37, 38], 2) prepositions, e.g., 为了 ‘for’, 3) homographs that are obviously ambiguous in pronunciation and in meaning, e.g., 穿着 ‘outfit’ as a noun or ‘wearing’ as a verb, and 4) non-words, e.g., 那是 ‘that is’. In the end, the final word sample consisted of 9,877 two-character words.

### 2.3 Procedure

To collect concreteness ratings, we consulted previous rating studies conducted in English, including Paivio [1] and a more recent study by Brysbaert et al. [20], as well as studies conducted in Mandarin Chinese [31, 32]. In keeping with prior research, the instructions of the present study first illustrated with examples concrete words versus abstract words. Specifically, a word is concrete if we learn about its referent mainly through sensory input e.g., *apple*, whereas a word is abstract if we learn about its referent mainly through language input, e.g., *soul*. It was further pointed out to the participants that word concreteness/abstractness varies on a continuous scale with some words falling in-between the two extremes. Participants were asked to indicate their assessment on a 5.0 numerical scale provided below each word, where “1” = “very concrete” and “5” = “very abstract”. To increase validity of the ratings, an additional option “N” was also provided to participants when they felt that they did not know the meaning of the word [20].

The 9,877 two-character words were divided into 40 lists of 246–247 words, roughly matching on word frequency. Each participant was randomly assigned to a questionnaire, including a list of 246–247 words and demographic questions attached to the end of the word list. The words were presented in a random order to each participant. The demographic questions were presented in a fixed order, asking participants to report gender, age, education level, first language, and the place where they spent most time of the first seven years of their lives [20].

We required participants to provide a response to each word on the list. However, they were free to withdraw at any time that they chose to do so. It took the participants on average 25 minutes to complete the word list and the demographic questions at the end.

## 3. Results

### 3.1 Data screening

We generated the frequencies of all possible responses, including 1–5 and the response “N”, representing “I don’t know the word”. First, we removed 13 (1.1%) participants who had 15% or more “N” responses as their vocabulary knowledge might be too limited to make credible concreteness/abstractness assessments [32]. Next, we removed 4 (0.4%) participants who had 85% or more same responses (210 out of 247 words) across the entire word list as such a low variation in one’s responses suggested noncompliance with the instructions [31]. Finally, we removed 42 (3.7%) participants whose ratings correlated poorly (< .10) with the rest of the participants assigned to the same word list [20]. As indicated earlier, these criteria resulted in the removal of a total of 59 participants, 5.2% of the original 1,140 participants.

Before we counted the number of valid ratings and calculated mean concreteness rating and standard deviation for each word, we removed all “N” responses (n = 4550), 1.7% of the total ratings from the 1081 participants. As a result, the total number of valid ratings was 262,374, and the number of valid ratings for each word ranged from 16 to 36. Words with 20 or more valid ratings accounted for 99.7% of the entire list of 9,877 words. Only 34 words (.3%) had 19 or less valid ratings. In the end, we found that inter-rater reliability for each of the 40 word lists was highly desirable, with Cronbach’s alphas ranging from .91 to .97, with a mean of .95 (SD = .01). Table 1 presents descriptive statistics of the present ratings.

**Table 1:** Summary of concreteness ratings.

N		9877
Mean		2.68
Median		2.76
Mode		3.00
Std. deviation		.78
Minimum		1.04
Maximum		4.56
Percentile	25	2.03
	50	2.76
	75	3.32

## 3.2 Data analysis

### 3.2.1 Correlation between the present and past concreteness ratings

**Fig 5:**
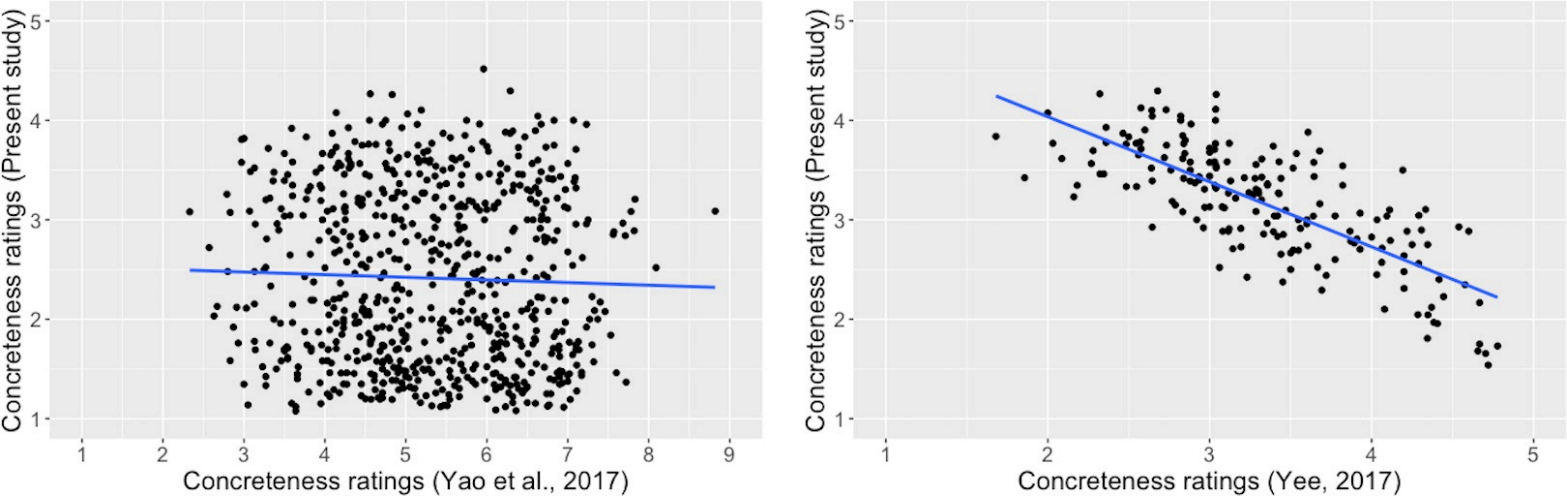
a. Correlation of the present concreteness ratings and concreteness ratings by Yao et al. [31], *r* = -.04, *p* = .34. (in Yao et al. [31], “1” represented “very abstract”, and “9” represented “very concrete”; in the present study, “1” represented “very concrete”, and “5” represented “very abstract”.). b. Correlation of the present concreteness ratings and concreteness ratings by Yee [32], *r* = -.75, *p* < .0001. (in Yee [32], “1” represented “very abstract”, and “5” represented “very concrete”; in the present study, “1” represented “very concrete”, and “5” represented “very abstract”.).

As indicated earlier, Yao et al. [31] and Yee [32] had only 26 words in common, and their assessments of concreteness about these words did not appear to be in alignment. We examined to what extend our ratings would converge with each of these two sets of ratings. Our word list had 693 and 184 words in common with Yao et al. [31] and Yee [32], respectively. Analyses showed no correlation of the current concreteness ratings with Yao et al., *r* (693) = -.04, *p* = .34, but a strong correlation with Yee, *r* (184) = -.75, *p* < .0001 (Fig 5A and 5B).

**Fig 6:**
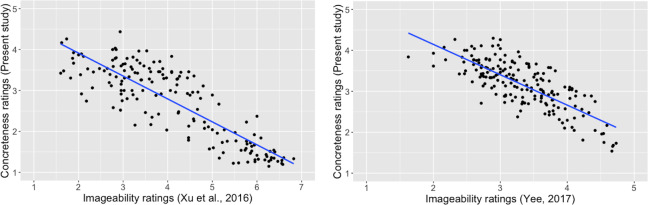
a. Correlation of the present concreteness ratings and imageability ratings by [34], *r* = -.85, *p* < .0001. (in [34], “1” represented “not imageable”, and “7” represented “highly imageable”; in the present study, “1” represented “very concrete”, and “5” represented “very abstract”.). b. Correlation of the present concreteness ratings and imageability ratings by Yee [32], *r* = -.77, *p* < .0001. (in Yee [32], “1” represented “difficult to form an image”, and “5” represented “easy to form an image”; in the present study, “1” represented “very concrete”, and “5” represented “very abstract”.).

Past research has shown that concreteness and imageability are two different, but closely associated constructs, and ratings of concreteness and imageability are highly correlated (e.g., [22] in French; [24], in Spanish; [29], in Chinese; Paivio, Yuille, & Madigan [39], in English; [25], in Portuguese). To further verify the validity of the current ratings, we therefore examined correlations of our concreteness ratings with imageability ratings retrieved from past studies. Both Yao et al. [31] and Yee [32] had collected imageability ratings. Xu, Kang, and Guo [40] also collected imageability ratings for a set of two-character Chinese words stimuli. Correlation coefficients between concreteness and imageability ratings are presented in Table 2. Most importantly, the present concreteness ratings were well-correlated with both imageability ratings by Xu et al. [34] and imageability ratings by Yee [32], supporting the validity of the current concreteness ratings (Fig 6A and 6B).

**Table 2:** Correlations of concreteness and imageability ratings between studies.

	1	2	3	4	5
1 Concreteness (present study)					
N					
2 Concreteness [31]	-.036				
N	693				
3 Concreteness [32]	-.753***	.044			
N	184	26			
4 Imageability [34]	-.853***	.145	.871**		
N	165	51	8		
5 Imageability [31]	-.046	.783***	-.007	.233	
N	693	693^a^	26	51	
6 Imageability [32]	-.774***	.032	.880***	.486	.032
N	184	26	184^a^	8	26

** *p* < .005 (two-tailed).

*** *p* < .0001 (two-tailed).

^a^ Only words on our list were included so that comparisons of correlation coefficients were more meaningful.

We investigated possible reasons for the lack of correlation between the current ratings and Yao et al. [31]. First, for the concreteness rating task, Yao et al. seemed to instruct participants to think of not only mental images associated with the to-be-rated word but also scenarios in which the word could occur. (The literal translation of the concreteness rating instruction is “concreteness is the degree to which one can think of an image or a scenario in reality.”) Therefore, their operational definition for concreteness seemed to be different from most studies that traditionally defined concreteness as a property related to sensorimotor experiences. Furthermore, according to Barsalou and Wiemer-Hastings [6], concrete concepts are mainly tangible objects and properties, whereas abstract concepts are represented as situations. Thus, the instruction containing the word “scenario” (which can also be translated as “circumstance” or “situation”) might have obfuscated the differences between concrete concepts and abstract concepts, resulting in reduced correlations of their concreteness ratings with ratings collected from other studies. For example, in Yao et al., both “etiquette” and “discipline” were rated more concrete than “traitor” (Fig 3), whereas it was the opposite in both Yee [32] and the present study.

**Fig 7:**
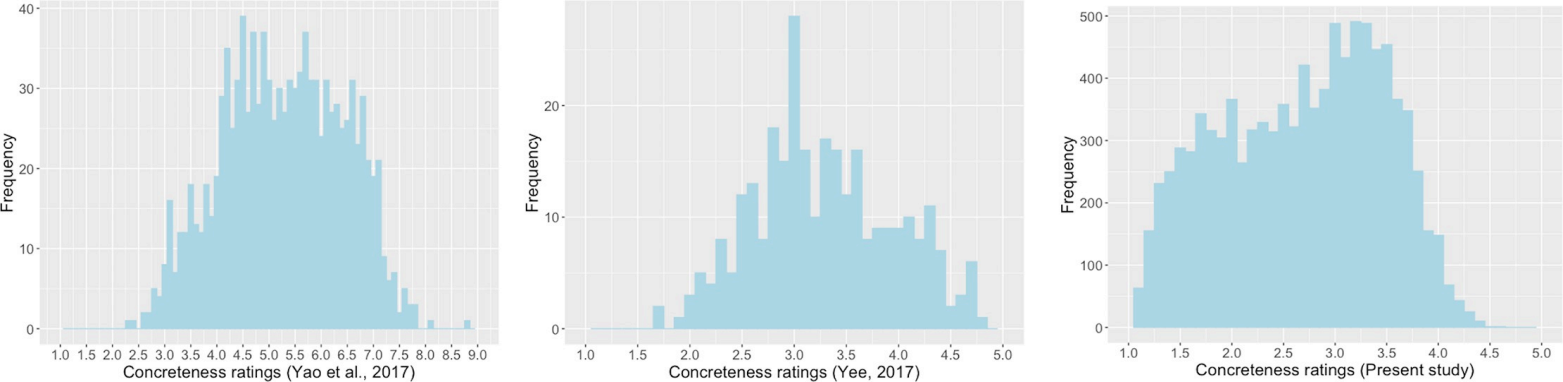
a. Distributions of concreteness ratings of Yao et al. [31]. b. Distributions of concreteness ratings of [32]. c. Distributions of concreteness ratings of the present study.

Second, relatively speaking, Yao et al.’s [31] word sample seemed to represent a restricted range of concreteness/abstractness. More specifically, there were a limited number of both highly concrete words and highly abstract words in the word sample of Yao et al. In comparison, the concreteness ratings for the sample of Yee [32] and the sample of the present study covered a more reasonable range. Although both lacked highly abstract words, highly concrete words seemed to have been better represented in both studies. Fig 7A, 7B and 7C show the range of word concreteness ratings for Yao et al. [31] in comparison with Yee [32] and the present study. (For a comprehensive discussion about the lack of highly abstract words as a common phenomenon in concreteness/abstractness research studies, please see Pollock [41]) That is, the lack of strong correlations between Yao et al. and the other studies might be partially due to the truncated range of concreteness/abstractness. The scatterplot (Fig 3) for the common words in Yao et al. [31] and Yee [32] illustrates the impact of truncated range on correlation.

**Fig 8:**
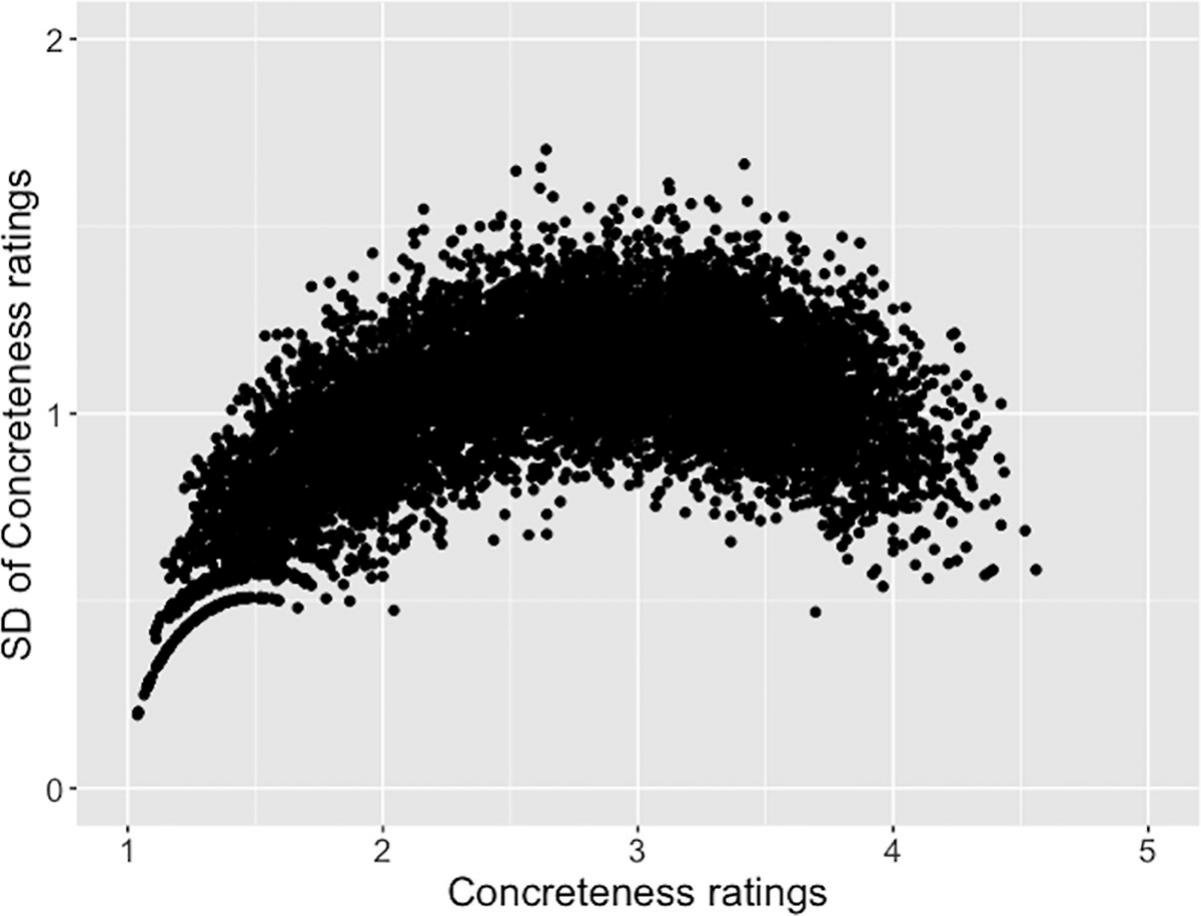
Standard deviation (SD) of concreteness ratings varies between the concrete extreme (1) and the abstract extreme (5) of the continuum.

As discussed earlier, words that fall in the middle of the concreteness-abstractness continuum must have a greater variability in perceived concreteness or abstractness. That is, individual differences in ratings about the referents of these words are likely more pronounced relative to the referents of words close to the two extremes. With the current ratings, we generated Fig 8, which supports this notion that rating variability changes along the rating scale.

More specifically, words close to the concrete end of the scale had the smallest variability in ratings. That is, raters were generally in agreement with regard to words that denote concrete entities (e.g., *egg* and *goat*). Next, there was some variability in perceived abstractness with regard to words close to the abstract end of the scale. These appeared to be mainly words related to cognitive, affective, or spiritual experiences (e.g., *suppose*, *ideal*, *emotion*, and *fate*). Lastly, words in the middle of the scale had the highest variability. The most prominent ones included concepts related to afterlife (e.g., *hades* and *zombie*) and concepts of specific subject areas (e.g., *physics* and *coefficient*), reflecting differences among people in views about life after death and differences in training and expertise.

### 3.2.2 Relations with word frequency and age-of-acquisition

**Fig 9:**
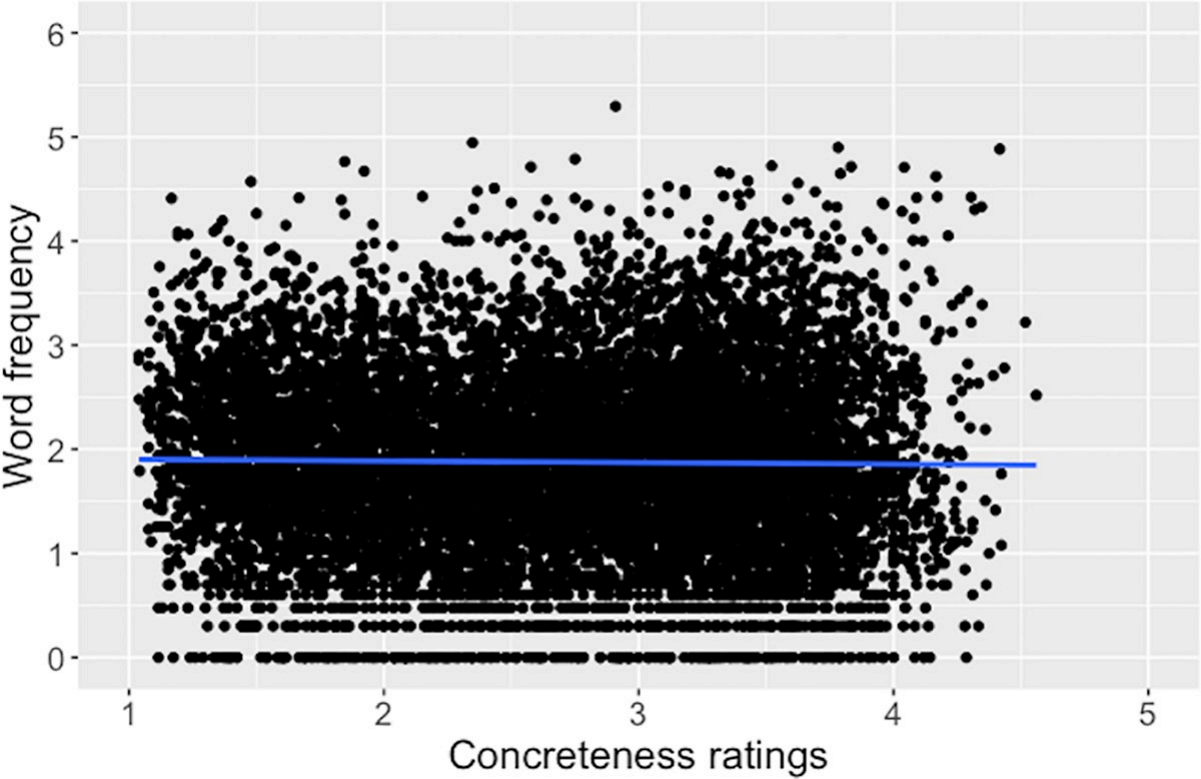
Correlation of the present concreteness ratings and word frequency [36], *r* = -.01, *p* = .17.

With our word sample, we explored the relations of concreteness with both word frequency and age-of-acquisition (AoA; [42]). Word frequency was retrieved from the SUBTLEX-CH corpus [36]. Table 3 presents the results. There was not a correlation between concreteness and word frequency (Fig 9). That is, concrete words do not appear more or less frequent than abstract words in the corpus.

**Table 3:** Correlations of concreteness ratings, word frequency, age-of-acquisition (AoA), zRT, and error rate.

	1	2	3	4
1 Concreteness				
N				
2 Word frequency (log)	-.014			
N	9877			
3 AoA	.382***	-.349***		
N	6000	6000		
4 zRT	.097***	-.633***	.381***	
N	9875	9875	6000	
5 Error rate	.100***	-.443***	.259***	.709***
N	9875	9875	6000	9875

*** *p* < .0001 (two-tailed).

**Fig 10:**
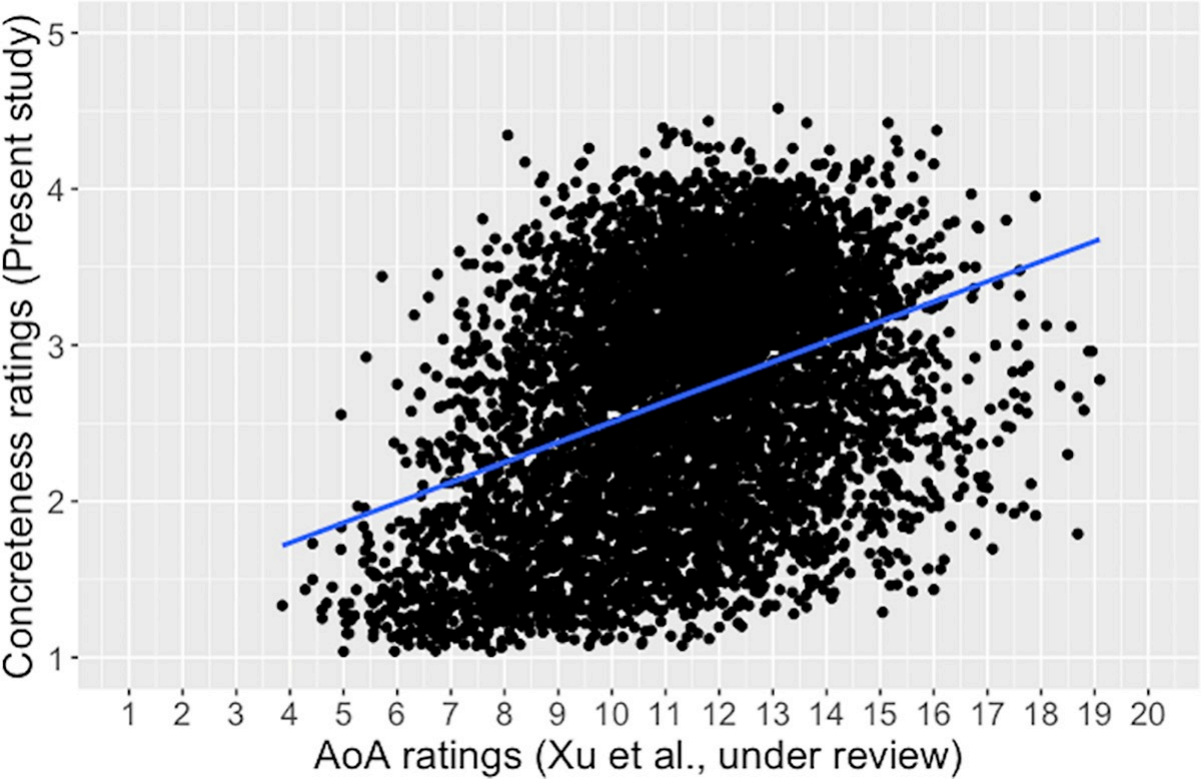
Correlation of the present concreteness ratings and age of acquisition (AoA; Xu et al. [43]), *r* = .38, *p* < .0001.

We retrieved AoA ratings from Xu, Li, and Guo [43]. There were in total 6,000 words in common between the two word samples. We ran a correlation analysis to verify the common notion that concrete words tend to be learned earlier relative to abstract words. There was indeed such a correlation (Table 3), albeit moderate in strength. Fig 10 shows that, although word abstractness in general increased with age, there appeared to be a wave of concentrated input of abstract words during the years of formal elementary and secondary education, which seemed to reflect the typical trajectory of vocabulary development for the majority of the raters in this study.

### 3.2.3 Relations with speed and accuracy of lexical decision

Finally, we examined the potential concreteness effect on lexical processing. Reaction times (RT) and error rates were retrieved from the MELD-SCH [35]. This database provided values of these two measures for all but two words on our list. Specifically, in the database, the second character of the word “魁梧” (meaning *burly*) appeared to be mistaken with another character. The other word “致敏” (meaning *allergenic*) was missing RT due to a “zero” accuracy rate. Thus, some of the following analyses contained 9,875 words excluding these two words. In keeping with many previous studies, standardized scores (zRT) were used for the analysis [44, 45, 35]. The correlation coefficients presented in Table 3 suggested that there might be only a weak concreteness effect on zRT and error rate. In contrast, unsurprisingly, word frequency showed the strongest correlation with both zRT and error rate, and AoA had a moderate correlation with the two measures, which were consistent with research of the English language [45].

We further tested the predictive powers of these three variables for speed and accuracy of lexical decision. Regression analyses with a forward approach found both word frequency and AoA significantly predicted zRT and error rate of lexical decision. Concreteness emerged as a weak predictor for error rate, but not for zRT. Table 4 presents significant predictors in these analyses.

**Table 4:** Significant predictors in regression analyses of zRT and error rate on word frequency, AoA, and concreteness.

	Predictor	B	SE	Beta	t	p	ΔR^2^
zRT	Word frequency (log)	-.184	.004	-.515	-47.350	-∞	.342
	AoA	.024	.001	.201	18.530	< .0001	.036
Error rate	Word frequency (log)	-2.292	.093	-.315	-24.604	< .0001	.131
	AoA	.329	.034	.135	9.736	< .0001	.020
	Concreteness	.273	.094	.038	2.907	.004	.001

## 4. Discussion

The present study reported concreteness ratings for 9,877 two-character Chinese words retrieved from the MELD-SCH [35]. The ratings showed a strong correlation with one of the two existing sets of ratings for two-character Chinese words [32], and were further validated through correlation analysis with imageability ratings. The lack of significant correlation between the present ratings and the other set of previously available ratings [31] appeared to be attributable to different rating instructions to assess word concreteness and possibly the truncated range of the word sample in Yao et al.

This study found that the amount of individual differences in perceived concreteness/abstractness varied along the concreteness-abstractness continuum. Words close to the concrete extreme of the continuum showed the highest level of agreement in concreteness ratings among raters. Typical examples were words referring to tangible entities such as animals and food. As a result, the mean concreteness/abstractness ratings of these words approximated to the extreme value (1) of concreteness on the scale (Fig 8). On the other hand, words close to the abstract extreme of the continuum showed some variability in ratings. Representative examples were words describing mental states and processes. Consequently, compared to concrete words, there appeared to be a scarcity of extremely abstract words based on the mean ratings of concreteness/abstractness. Lastly, words in the middle portion of the continuum showed the highest level of variability in ratings. Most distinctive examples were words reflecting different views about afterlife and different areas of training and specialty among raters.

These findings seem to have an important implication for word sampling in research. Specifically, for studies that aim to detect or evaluate concreteness and abstractness effects, the presence and the size of these effects may at least partially depend on the degree of contrasts in mean concreteness/abstractness ratings of word stimuli. That is, a greater contrast in ratings of concrete stimuli versus abstract stimuli naturally increases the detecting power of the study. In reality, however, through a close examination of word stimuli utilized in past research studies, Pollock [41] found many word samples contained highly concrete words, but relatively abstract words, i.e., words sampled from the middle part of concreteness/abstractness scale, which may increase the chance of Type II error. Further, as just indicated, stimuli sampled from different portions of the rating scale differ in variability of perceived concreteness/abstractness, and thus differ in typicality and even utility to represent the experimental conditions (concrete versus abstract) that they are assigned to represent. This is particularly relevant given the fact that concreteness and imageability are highly correlated, and the latter is often needed to be controlled in research on concreteness and abstractness effects. In this case, all word stimuli are likely sampled from the middle portion of the rating scale, and thus vary greatly in perceived concreteness/abstractness from person to person. Consistent with Pollock’s argument, findings of the present study suggest that, in order to effectively detect the concreteness or abstractness effect, it is not only necessary to ensure sufficient contrasts in concreteness/abstractness ratings of word stimuli between experimental conditions, but also to keep variability of concreteness/abstractness ratings at a low level in order to sample stimuli most representative of their respective experimental conditions. In addition, Pollock’s experiments revealed another important factor to take into account while investigating the concreteness and abstractness effects. Kousta et al. [8] argued that affective experiences play a statistically predominant role in the representation of abstract concepts. Pollock showed that, after incorporating word valence, Experiment 3 was able to detect the concreteness effect which was missing when valence was not taken into account (Experiments 1 and 2).

The present study also examined the relations of word concreteness with word frequency, AoA, and efficiency of lexical processing. First, it was found that, when the full range of word concreteness/abstractness is considered, it did not appeared to be associated with word frequency. That is, concrete and abstract words seem comparable in how frequently they are invoked in our verbal discourses to accomplish effective communications. Second, there was only a moderate correlation between concreteness and AoA, reflecting the typical trajectory of vocabulary development and the impact of formal education on the growth of one’s mental lexicon. Specifically, in general, as we age, we learn words that are increasingly more abstract. Our repertoire of abstract words however grows exponentially during the years of formal schooling, especially the later part of elementary education and the early part of secondary education (Fig 10). Lastly, relative to word frequency and AoA, concreteness appeared to have a limited effect on the efficiency of lexical processing. That is, consistent with past findings, the commonly held notion that word concreteness is associated with more efficient processing may be partially due to word frequency and possibly limited to comparison of specific categories of words under specific experimental designs [46, 47, 48, 49, 50, 51]. In addition, the findings in this study about the effect of concreteness, or lack thereof, on lexical decision are consistent with findings in Dutch [52]. Concreteness, as a semantic dimension, may only play a minor role in a lexical decision task, which entails a relatively shallow level of processing [53] relative to semantic judgment tasks such as valence categorization. In contrast, lexical variables, such as word familiarity (i.e., subjective familiarity with a word; [54]) and word prevalence (i.e., number of people in the population who know the word; Brysbaert et al. [52] seem to be more prominent in relation to the efficiency of lexical decisions. These findings, together with the clinical significance of word concreteness/abstractness discussed in the introduction, highlight word concreteness/abstractness as a unique construct that is worthy of further investigation.

In summary, this study made available concreteness/abstractness ratings for a large set of two-character Chinese words, which makes it possible not only to further expand future theoretical and clinical research on the concreteness/abstractness effects in the Chinese language, but also to take into account this important conceptual feature when conducting cross-language research such as bilingualism and translation studies between Chinese and other languages, for which a large set of word concreteness/abstractness ratings is also available.

## Supporting information

S1 Data (RAR)

S2 Data (PDF)

S1 Appendix (DOCX)
