# Supplementary material for: Concreteness/abstractness ratings for two-character Chinese words in MELD-SCH
Source: PLoS One. 2020 Jun 22;15(6):e0232133. doi: 10.1371/journal.pone.0232133 (PMC7307783; doi:10.1371/journal.pone.0232133)
Supplement: S2 Data — (PDF) [file pone.0232133.s002.pdf]

问卷 A:

您的年级_____　　您的性别_____　　您的年龄_____　　您的专业_____

同学：您好！

欢迎参加中国情绪材料数据库的验证性测评！请认真对下列词汇的四个维度进行评价，用 1 至 9 的数字以示阅读该词后心情感受的强弱程度。

愉悦度是指愉快或不愉快的情绪体验及其强度，其得分从 1（非常不愉快）到 9（非常愉快）之间变化。**唤醒度**指与情绪活动相伴随的机体唤醒程度，其得分从 1（不兴奋，非常没精神）到 9（非常提神和兴奋）之间变化。**具体性**指能在现实中找到明确的形象或场景的程度，其得分从 1（找不到具体的形象，抽象的）到 9（有非常明确的形象，具体的）之间变化。**熟悉度**指阅读该词时感觉熟悉的程度，其得分从 1（生疏的，陌生的）到 9（熟悉的、常见的）之间变化。**注意，1 代表相应的感受最弱，9 代表相应的感受最强。**

举个例子："婚礼"这个词，读完后通常有比较愉悦的心情（愉悦度），心情比较激动（唤醒度），脑海里能够呈现出婚礼的场景（具体性），对该词也不陌生（熟悉度），那么这四个维度的评定等级通常都在 6 以上。而"事实"这个词，读完后感到平静，心情也比较平静，在现实中找不到这个词对应的形象，对该词不太陌生也不太熟悉，那么对该词四个维度的评定通常在 5 分左右。**读完一个词汇的感受是因人而异的，没有对错之分，因此评定的时候依据自己的第一感觉即可。**

Questionnaire A:

Your year_____　　Your gender_____　　Your age_____　　Your major_____

Dear Classmate:

Welcome to take part in the rating task of the Chinese emotional material database! Please carefully rate each word in each dimension given (valence, arousal, concreteness and familiarity), and use numbers 1 to 9 to express your feelings after reading the following words.

Valence is the extent to which the word makes you feel negative (sad, scared) or positive (happy, contented). **Arousal** is the extent to which the word makes you

feel calm (relaxed, bored) or excited (stimulated, agitated). **Concreteness** is rated according to how concrete/tangible a concept is in the real world. **Familiarity** is rated according to the level of familiarity when you read each word. For each word, you can choose one response among 9 levels, with 1 indicating very negative, very calm, highly abstract, and unfamiliar and 9 indicating very positive, very exciting, highly concrete, and familiar respectively.

For instance, If you think that *"婚礼 (wedding)"* has a very positive meaning, and makes you feel excited, conjuring up a specific image of certain situations in real world, and impresses you as familiar, the levels of rating on these respective dimensions are commonly more than five. If you think that *"事实 (fact)"* does not evoke either positive or negative feeling, or provoke any significant emotional arousal, or conjuring up any particular scene in your mind, nor are you very familiar with it, the levels of rating on these dimensions are commonly less than five".

Notice, there are no right or wrong answers. Please do not spend a lot of time thinking about your ratings, because your spontaneous reactions are of the greatest interest.

问卷 B:

您的年级：_____　　　您的性别_____　　　您的年龄_____　　　您的专业_____

同学：您好！

欢迎参加中国情绪材料数据库的评定！请认真对下列词汇的两个个维度进行评价，用 1 至 9 的数字以示阅读该词后心情感受的强弱程度。

表象性是指在你的脑海里，你能否容易地想象出这个词表达的形象。**语境获得性**是指你能否容易地想起这个词出现的特定语境或场景。用 1 到 9 的数字来表示该词在这两个属性上的难易程度，其得分从 1（非常难）到 9（非常容易）之间变化。**注意，1 代表相应的感受最弱，9 代表相应的感受最强。**

例如：对我来说，“婚礼”这个词，读完这个词后很容易在脑海里呈现一个婚礼的场景，因此表象性得分较高（7 左右）；而这个词也容易出现在文字或者现实生活中，也就是说我能够比较容易地想起这个词出现的环境（语境获得性 7 左右）。而“事实”这个词，读完后脑海里很难想象它表达的形象（表象性得分 1），也比较不容易想起这个词出现的语境或环境（语境获得性 2）。

读完一个词汇的感受是因人而异的，没有对错之分，因此评定的时候依据自己的第一感觉即可。

Questionnaire B:

Your year_____　　　Your gender_____　　　Your age_____　　　Your major_____

Dear Classmate:

Welcome to take part in the rating task of the Chinese emotional material database! Please carefully rate each word in each dimension given (valence, arousal, concreteness and familiarity), and use numbers 1 to 9 to express your feelings after reading the following words.

Imageability is rated according to how easy it is for each word to elicit a visual image of the concept the word indicates to them. **Context availability** is rated according to how easy it is to come up with a particular context or circumstance in

which the word might appear. For each word, you can choose one response among 9 levels, with 1 indicating difficult to image and difficult to think of a context, and 9 indicating easy to image and easy to think of a context respectively.

For example, " If when you read the word*"婚礼 (wedding)"*, you can very easily image a scene of the wedding in your brain, and the notion of a happy bride in the lawn would come immediately to our minds. In this case, the levels of rating on these dimensions usually are more than six. Conversely, If when you read the word "*事实 (fact) "* , you can very hardly image a scene of it in your mind, and it seems to difficultly be associated with some word when this word appears. In this case, the levels of rating on these dimensions usually are less than three even two.

Notice, there are no right or wrong answers, please not to spend a lot of time thinking about your ratings, because yours first considerations are of greatest interest.
