# Supplementary material for: Concreteness/abstractness ratings for two-character Chinese words in MELD-SCH
Source: PLoS One. 2020 Jun 22;15(6):e0232133. doi: 10.1371/journal.pone.0232133 (PMC7307783; doi:10.1371/journal.pone.0232133)
Supplement: S1 Appendix — (DOCX) [file pone.0232133.s003.docx]

Appendix: Words in common between [52] and [53]

| Chinese word | English translation |
| --- | --- |
| 才华 | talent |
| 法规 | law |
| 风光 | scenery |
| 福利 | welfare |
| 规律 | regularity |
| 纪律 | discipline |
| 见解 | opinion |
| 礼仪 | etiquette |
| 利润 | profit |
| 叛徒 | traitor |
| 品味 | taste |
| 前途 | future |
| 人格 | personality |
| 人情 | human relationship |
| 事态 | situation |
| 誓言 | oath |
| 收益 | income |
| 手艺 | craft |
| 守则 | code |
| 思维 | thinking |
| 岁月 | time |
| 条例 | regulation |
| 文明 | civilization |
| 效率 | efficiency |
| 信念 | belief |
| 真理 | truth |
